# Methylation profiling of paediatric pilocytic astrocytoma reveals variants specifically associated with tumour location and predictive of recurrence

**DOI:** 10.1002/1878-0261.12062

**Published:** 2018-07-06

**Authors:** Alexandra Sexton‐Oates, Andrew Dodgshun, Volker Hovestadt, David T. W. Jones, David M. Ashley, Michael Sullivan, Duncan MacGregor, Richard Saffery

**Affiliations:** ^1^ Murdoch Childrens Research Institute The Royal Children's Hospital Parkville Australia; ^2^ Department of Paediatrics The University of Melbourne Parkville Australia; ^3^ Children's Cancer Centre The Royal Children's Hospital Parkville Australia; ^4^ Department of Paediatrics University of Otago Christchurch New Zealand; ^5^ Division of Molecular Genetics German Cancer Research Centre (DKFZ) Heidelberg Germany; ^6^ Division of Pediatric Neurooncology German Cancer Research Centre (DKFZ) Heidelberg Germany; ^7^ School of Medicine Deakin University Waurn Ponds Australia; ^8^ Department of Anatomical Pathology The Royal Children's Hospital Parkville Australia; ^9^ Department of Pathology The University of Melbourne Parkville Australia

**Keywords:** DNA methylation, pilocytic astrocytoma

## Abstract

Childhood pilocytic astrocytomas (PA) are low‐grade tumours with an excellent prognosis. However, a minority, particularly those in surgically inaccessible locations, have poorer long‐term outcome. At present, it is unclear whether anatomical location in isolation, or in combination with underlying biological variation, determines clinical behaviour. Here, we have tested the utility of DNA methylation profiling to inform tumour biology and to predict behaviour in paediatric PA. Genome‐wide DNA methylation profiles were generated for 117 paediatric PAs. Using a combination of analyses, we identified DNA methylation variants specific to tumour location and predictive of behaviour. Receiver‐operating characteristic analysis was used to test the predictive utility of clinical and/or DNA methylation features to classify tumour behaviour at diagnosis. Unsupervised analysis distinguished three methylation clusters associated with tumour location (cortical, midline and infratentorial). Differential methylation of 5404 sites identified enrichment of genes involved in ‘embryonic nervous system development’. Specific hypermethylation of *NEUROG1* and *NR2E1* was identified as a feature of cortical tumours. A highly accurate method to classify tumours according to behaviour, which combined three clinical features (age, location and extent of resection) and methylation level at a single site, was identified. Our findings show location‐specific epigenetic profiles for PAs, potentially reflecting their cell type of origin. This may account for differences in clinical behaviour according to location independent of histopathology. We also defined an accurate method to predict tumour behaviour at diagnosis. This warrants further testing in similar patient cohorts.

**Abbreviations**
AUC: area under the curve
CNS: central nervous system
DMP: differentially methylated probe
DNA: deoxyribonucleic acid
EFS: event‐free survival
FFPE: formalin‐fixed paraffin‐embedded
HM450K: Illumina Infinium HumanMethylation450 BeadChip Array
HREC: Human Research Ethics Committee
MAPK: mitogen‐activated protein kinase
MRI: magnetic resonance imaging
PA: pilocytic astrocytoma
PC: principal component
RCH: Royal Children's Hospital
ROC: receiver‐operating characteristic
SEQUENOM: SEQUENOM MassARRAY
SNP: single nucleotide polymorphism

## 1 Background

Pilocytic astrocytoma (PA), a WHO‐grade I glial tumour, is the most common central nervous system (CNS) tumour in children, accounting for 15% of all diagnoses (Ostrom *et al*., 2015). Approximately 5–10% of PAs arise in children with neurofibromatosis type 1, most occurring in the optic pathway (Marko and Weil, 2012). In contrast, sporadic PAs arise most commonly in the cerebellum, followed by the supratentorial region, optic pathway and hypothalamus, brainstem and spinal cord (Burkhard *et al*., 2003; Collins *et al*., 2015). PAs grow slowly and rarely transform to higher grades or metastasise (Collins *et al*., 2015); however, the possibility of local recurrence remains (particularly after incomplete surgical resection) (Dodgshun *et al*., 2016; Gnekow *et al*., 2012; Youland *et al*., 2013). PA in childhood has an excellent prognosis, with a 10‐year overall survival rate of over 90% (Bandopadhayay *et al*., 2014; Collins *et al*., 2015; Ohgaki and Kleihues, 2005); however, age (< 2 years), location (extracerebellar) and incomplete resection are associated with poorer outcome (Bandopadhayay *et al*., 2014; Collins *et al*., 2015). Whilst overall survival is an important measure of treatment outcome, long‐term morbidity and the quality of survival is a crucial consideration when deciding upon treatment.

Given that location strongly influences the degree of resection, treatment approach, chance of recurrence and associated treatment‐related morbidity, understanding possible location‐specific biological behaviour is important (Marko and Weil, 2012; Zhang *et al*., 2013). Whilst there is no difference in the frequency of copy number variations according to PA location, there is evidence to show that tumour location is associated with specific MAPK pathway alterations (Jones *et al*., 2006; Pfister *et al*., 2008). For example, *KIAA1549‐BRAF* fusions are more common in posterior fossa tumours (up to 90%), compared to supratentorial tumours (33–59%), and *BRAF* V600E mutations are more frequent in supratentorial tumours (~ 18%) than those in the posterior fossa (~ 3%) (Bergthold *et al*., 2015; Horbinski, 2013; Zhang *et al*., 2013). These location‐specific alterations are also seen in gene expression analyses that show significant differences between supratentorial and infratentorial PAs and between hypothalamic/chiasmatic and posterior fossa PAs (Bergthold *et al*., 2015; Sharma *et al*., 2007; Tchoghandjian *et al*., 2009).

Whilst the role of the MAPK pathway in PA is well known, less is known about other ‘layers’ of molecular disruption, including DNA methylation. DNA methylation analysis has identified clinically distinct subtypes of CNS tumours including medulloblastoma, ependymoma, anaplastic astrocytoma and glioblastoma (Northcott *et al*., 2015; Pajtler *et al*., 2015; Sturm *et al*., 2012; Wiestler *et al*., 2014); therefore, it is relevant to investigate its role in PA. Recently, Lambert *et al*. (2013) identified genome‐wide DNA methylation differences between 62 supratentorial and infratentorial PAs with concurrent changes in gene expression.

In the current study, we aimed to build on this work, refining DNA methylation signatures of PAs in specific brain regions and exploring the potential of DNA methylation to predict tumour recurrence. Importantly, whilst most of the previous work has focussed on infratentorial versus supratentorial location alone, we have compared three location groups of infratentorial, midline and cortical origin. Grouping tumours in this way allows investigation of supratentorial midline tumours separately to those of the cerebral cortex, a clinically important consideration given the difficulty of surgical resection and high rates of recurrence in midline tumours (Tchoghandjian *et al*., 2009).

## 2 Materials and methods

### 2.1 Tumour samples

**Figure 1:**
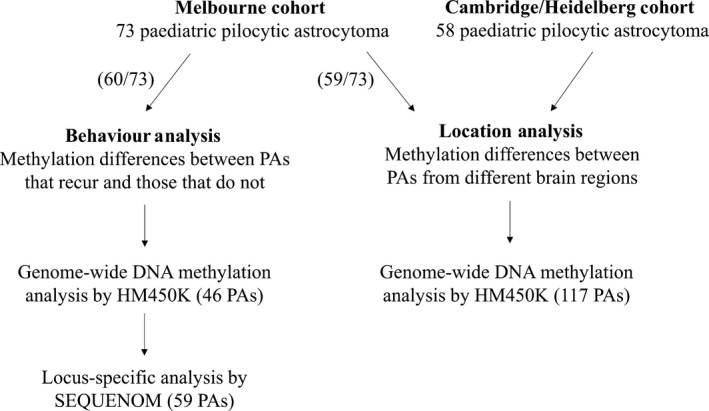
Study overview. Data were generated from two cohorts for the analysis of tumour location differences in DNA methylation profile; this included 59 tumours from the Melbourne cohort (with sufficient DNA for analysis by the HM450K array) and 58 tumours collected from Cambridge/Heidelberg. Tumour behaviour analysis was restricted to 60 samples from the Melbourne cohort (those with sufficient clinical information); of those, 46 had sufficient DNA for analysis by the HM450K array and 59 had sufficient DNA for analysis by SEQUENOM.

Tumour samples from two cohorts were used in the study: the Melbourne cohort and the Cambridge/Heidelberg cohort. The first comprised formalin‐fixed, paraffin‐embedded (FFPE) tumour tissue from 73 children diagnosed with PA at the Royal Children's Hospital (RCH), Melbourne, between 1998 and 2014. Diagnosis of PA was confirmed by histological assessment by a paediatric anatomical pathologist (D.M.). None had undergone chemotherapy or radiotherapy prior to resection. Recurrence status, defined as the presence or absence of increased growth or regrowth at the original resection site reported in any surveillance magnetic resonance imaging (MRI) scans during a five‐year period from diagnosis, was available for 60 patients. This study was approved by the RCH Human Research Ethics Committee, HREC #34040A and #34049C. The Cambridge/Heidelberg cohort comprised 58 snap‐frozen paediatric PA tissues from tissue banks in Cambridge, United Kingdom and Heidelberg, Germany. The features of this cohort have been previously described (Lambert *et al*., 2013). An overview of the samples used for each analysis is shown in Fig. 1.

### 2.2 DNA extraction

Haematoxylin and eosin‐stained slides from the Melbourne cohort were reviewed by D.M. to ensure tumour content of ≥ 90%. Between 2 and 4 10‐μm FFPE sections were cut and deparaffinised with xylene, and DNA was extracted using the QIAamp DNA FFPE Tissue Kit (QIAGEN, Dusseldorf, Germany). DNA was extracted from snap‐frozen tissue in the Cambridge/Heidelberg cohort, with a tumour cell content of ≥ 70%, as previously described (Lambert *et al*., 2013).

### 2.3 Genome‐wide DNA methylation detection

Melbourne cohort: Genome‐wide DNA methylation was measured on bisulfite‐converted genomic DNA using the Illumina Infinium HumanMethylation450 BeadChip Array (HM450K) at ServiceXS (the Netherlands). Cambridge/Heidelberg cohort: Genome‐wide DNA methylation was measured on bisulfite‐converted genomic DNA using the HM450K array at the German Cancer Research Centre Genomic and Proteomics Core Facility. Raw methylation data from both cohorts were combined for analysis. Data were processed using the *lumi* and *minfi* packages for R and normalised using SWAN (Maksimovic *et al*., 2012). Probes on the X and Y chromosomes, associated with single nucleotide polymorphisms (minor allele frequency > 1%), or which failed in one or more samples were removed, leaving data for 422 877 probes common to all samples for subsequent analysis. Probable *BRAF* fusion status was predicted by manual inspection of copy number profiles generated from HM450K data by the *conumee* package for R. Control brain: raw HM450K array IDAT files for 14 snap‐frozen control adult brain tissues were obtained from D.T.W.J. Tissues were from the cerebellum, hypothalamus and cerebral hemispheres. Data were processed as above, which yielded data for 453 805 probes.

### 2.4 Locus‐specific DNA methylation detection

Approximately 10 ng of genomic DNA was bisulfite‐converted using the MethylEasy Xceed Kit (Human Genetic Signatures, Randwick, Australia). Methylation assays for the validation of HM450K data were designed using epidesigner software (http://www.epidesigner.com) that covered target probe regions of interest. Primer sequences and annealing temperatures are shown in Table S1. Predicted cleavage patterns were determined using the *MassArray* package for R. DNA methylation was detected using the SEQUENOM MassARRAY platform and EpiTYPER software (SEQUENOM). All samples were amplified and assayed in triplicate, and the mean methylation value was calculated after discarding outliers (deviation of ± 10% from the median) as previously described (Ollikainen *et al*., 2010).

### 2.5 BRAF mutation detection

Melbourne cohort: samples were analysed for the presence of the *BRAF* V600E mutation by sequencing as previously described (Myung *et al*., 2012); primer sequences and annealing temperatures are shown in Table S2. Cambridge/Heidelberg cohort: samples were analysed for the presence of *BRAF* mutations as previously described (Lambert *et al*., 2013).

### 2.6 Immunohistochemistry

Immunohistochemistry was performed on paraffin‐embedded sections using the BenchMark ULTRA system (Roche, Castle Hill, Australia). Anti‐NR2E1 antibody was obtained from Abcam (Cambridge, UK) and diluted 1 : 250 with Ventana antibody dilution buffer (Roche).

### 2.7 Data analysis

Multidimensional scaling plots, which encompass a Euclidean distance determination of relatedness, were generated from all methylation values. Supervised analysis was performed using the *WGCNA* and *limma* packages for R. Linear regression analysis was used to compare methylation between groups according to tumour location, taking into account the following covariates: study cohort, HM450K chip number and patient age, as identified by principal component (PC) analysis as contributing significantly (*P* < 0.05) to overall variation. Linear regression analysis was also used to compare methylation between groups according to tumour behaviour, taking into account the following covariates: HM450K chip number, tumour location, patient age and patient sex, as identified by PC analysis as contributing significantly (*P* < 0.05) to overall variation. The Benjamini–Hochberg FDR method (Benjamini and Hochberg, 1995) was used to adjust for multiple testing when defining statistically differentially methylated probes (DMPs). Methylation distribution at individual genes and sites according to location or behaviour was assessed using the Mann–Whitney *U*‐test. Correlations of SEQUENOM methylation values with HM450K values were assessed using Pearson's correlation coefficient. Kaplan–Meier curves were produced using the *survival* package for R. Comparison of event‐free survival (EFS) times between groups was made using the two‐tailed log‐rank test. An ‘event’ was defined as increased growth, or regrowth at the original resection site reported in any surveillance MRI scans during a five‐year period from diagnosis. Receiver‐operating characteristic (ROC) analysis was performed to identify whether clinical features, alone or in combination, could accurately classify tumours into ‘recurred’ and ‘no recurrence’ behaviour categories. Ability to classify correctly can be inferred by the area under the curve (AUC) value, which ranges from 0 to 1.0. AUC values were determined using ROC analysis using the *pROC* package for R.

### 2.8 Pathway analysis

A list of 1806 individual genes, corresponding to 5404 probes (adjusted *P* ≤ 0.01), identified as differentially methylated between tumour locations, was interrogated using a core analysis approach in the Ingenuity Pathway Analysis package (http://www.ingenuity.com).

## 3 Results

In total, 117 paediatric PAs were subject to genome‐wide DNA methylation analysis. These comprised tumours from eight infants (0–18 months), 87 children (19 months–11 years) and 22 adolescents (12–18 years). The majority of tumours were infratentorial (82), with 23 midline and 12 cortical (Table S3). Of tumours, 71% had a *BRAF* fusion and 8% had a *BRAF* V600E mutation. There were no significant differences in patient sex, age or tumour location between cohorts (all *P* > 0.05, Fisher's exact test).

### 3.1 DNA methylation profiling identifies three PA subgroups according to tumour location

**Figure 2:**
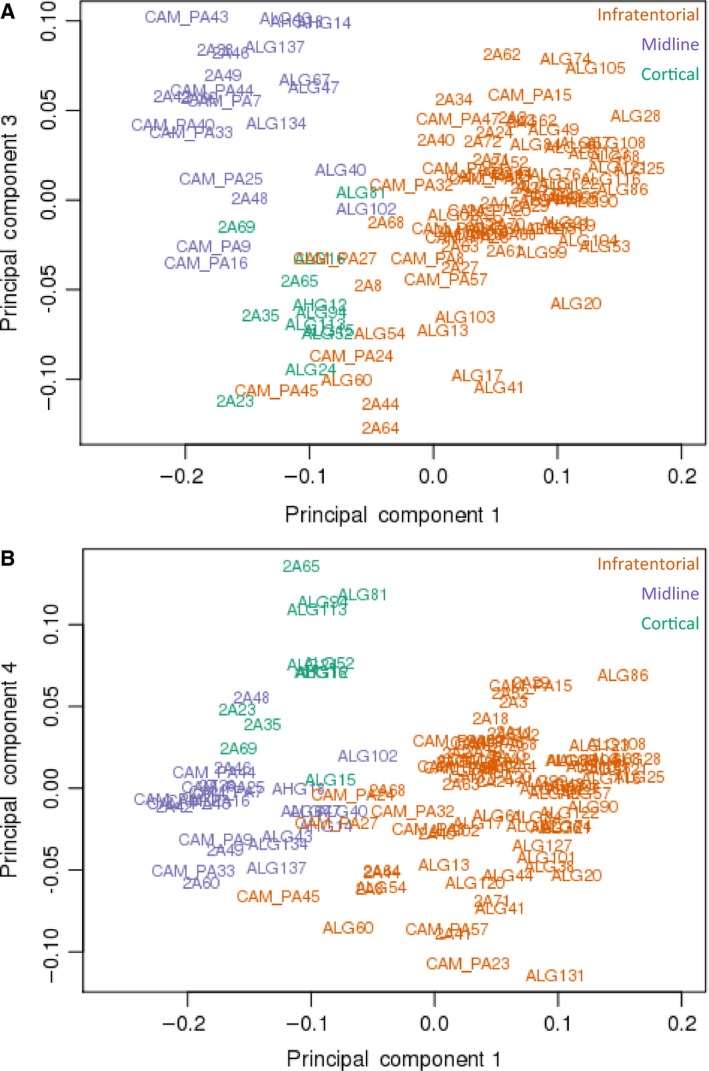
(A) Beta values for 422 877 HM450K probes were used to produce an MDS plot of sample relatedness over principal components 1 and 3. This revealed two methylation clusters with overlap between infratentorial and cortical tumours, and that tumour location is an important source of variation in DNA methylation profile. (B) Beta values for 422 877 HM450K probes were used to produce an MDS plot of sample relatedness over principal components 1 and 4. This revealed two methylation clusters with overlap between infratentorial and midline tumours, and that tumour location is an important source of variation in DNA methylation profile.

**Figure 3:**
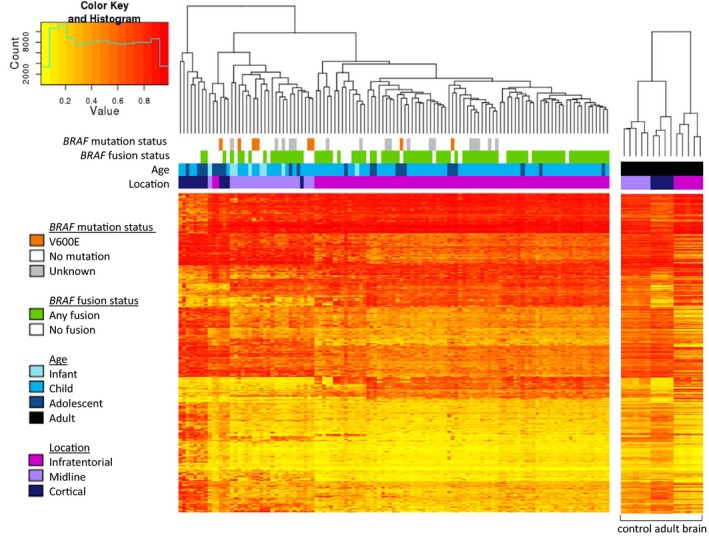
Individual samples are plotted on the *x*‐axis, and individual probes on the *y*‐axis. Completely unmethylated probes (beta value of 0) are represented by yellow, and completely methylated probes (beta value of 1) are represented as red; values in between 0 and 1 are represented by a spectrum of colours from yellow and red. The branch dendrogram indicates the relatedness of samples by methylation; branches closer together are more similar than those further apart. The histogram depicts the distribution of methylation levels across all samples and probes; the beta value is plotted on the *x*‐axis and the number of probes on the *y*‐axis. The heatmap showed that the majority of tumours in each location group clustered together, with the exception of two infratentorial tumours and four cortical tumours that clustered with the midline tumours. Infratentorial and cortical tumours had widely disparate methylation levels across the 1000 probes, whilst midline tumours variably clustered with the infratentorial and cortical tumours. Over the 1000 probes, control adult brain clustered by region of origin.

Principal component analysis was used to define the major sources of variation within the data set. This revealed PC2 as primarily being due to study and/or sample source, which was taken into account in subsequent analyses. Multidimensional scaling analysis of PC1 vs PC3 and PC1 vs PC4 largely separated tumours according to anatomical location (Fig. 2A,B), confirming a distinct DNA methylation profile of each. Linear regression analysis identified 5404 DMPs (adjusted *P* ≤ 0.01) between the three different regions. Unsupervised hierarchical clustering using the top 1000 DMPs demonstrated that cortical and infratentorial tumours had widely disparate methylation profiles, whereas the midline group of tumours variously clustered with the infratentorial or cortical tumours (Fig. 3). A role for tissue of origin (as opposed to differences in PA tumours *per se*) in driving this effect is supported by clustering of control brain tissue from the same regions in adults, which clearly separated by tissue of origin and showed a similar methylation ‘pattern’ to the paediatric PA samples.

### 3.2 Differentially methylated genes are enriched for roles in embryonic nervous system development networks

**Figure 4:**
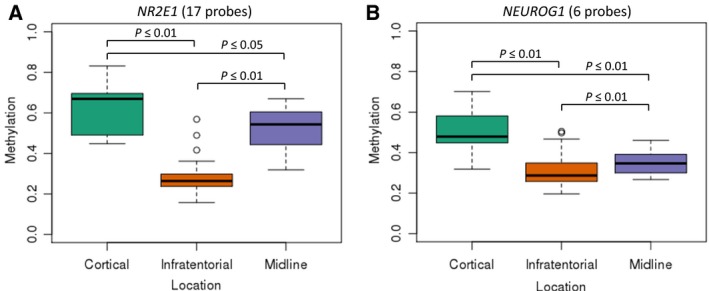
Box and whisker plots show median, interquartile range, lowest value within 1.5 × IQR, largest value within 1.5 × IQR and outliers for each location group. Median methylation level of cortical, midline and infratentorial tumours across (A) 17 probes in the NR2E1 gene body (cortical: 0.67, midline: 0.54, infratentorial: 0.26) and (B) six probes in the transcription start site of NEUROG1 (cortical: 0.48, midline: 0.35, infratentorial: 0.29).

Pathway analysis of 1806 genes associated with location‐specific DMPs (adjusted *P* ≤ 0.01) identified a significant enrichment for ‘embryonic nervous system development’ and ‘nervous system development and function’ networks. Of particular interest were genes associated with neuroglial development. Given their involvement in this pathway, *NR2E1* (nuclear receptor subfamily 2 group E) and *NEUROG1* (neurogenin 1) were chosen for further targeted investigation. *NR2E1* contained 17 DMPs located in the gene body, and *NEUROG1* contained six in the transcription start site. As shown in Fig. 4A,B, *NR2E1* and *NEUROG1* had significantly higher levels of methylation in cortical tumours relative to infratentorial and midline tumours (*P* ≤ 0.05). Infratentorial tumours also had significantly lower levels of methylation in *NR2E1* and *NEUROG1* when compared to midline tumours (*P* ≤ 0.01).

### 3.3 Differential methylation of NR2E1 is not associated with differential protein expression

**Figure 5:**
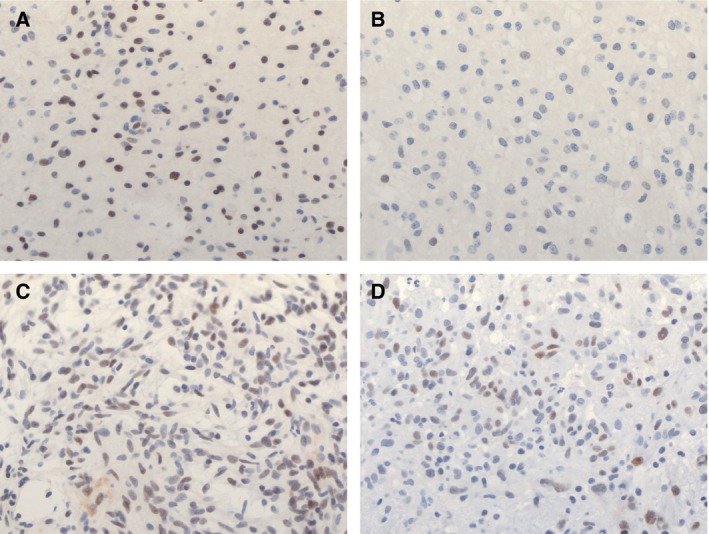
Immunohistochemical staining for NR2E1 in tumours from three different locations. (A) Dense region of staining seen within an infratentorial tumour and (B) sparse staining within the same tumour. (C) Staining pattern in a midline tumour. (D) Staining pattern in a cortical tumour. Results showed variable expression across tumours with no clear differences between tumours from different locations. Furthermore, there was considerable variation in expression within individual tumour tissues.

Immunohistochemical staining was performed on 59 PAs (Melbourne cohort) to detect expression of NR2E1. Results showed variable expression across tumours with no clear differences between tumours from different locations (Fig. 5). Furthermore, there was considerable variation in expression within individual tumour tissues (Fig. 5A,B). Control tissue (normal cortex) was stained consistently across all slides.

### 3.4 DNA methylation profiling reveals limited variation associated with tumour recurrence

A subset of 46 tumours from the initial 117 had sufficient data related to disease course to be included in an analysis of factors contributing to tumour recurrence. Within five years of diagnosis, 18 tumours had recurred, whilst 28 had not (Table S4). Unsupervised analysis of methylation data from probes contributing to the first two PCs of variation in the total data set (or any other specific PC combinations) did not separate cases according to tumour behaviour.

**Figure 6:**
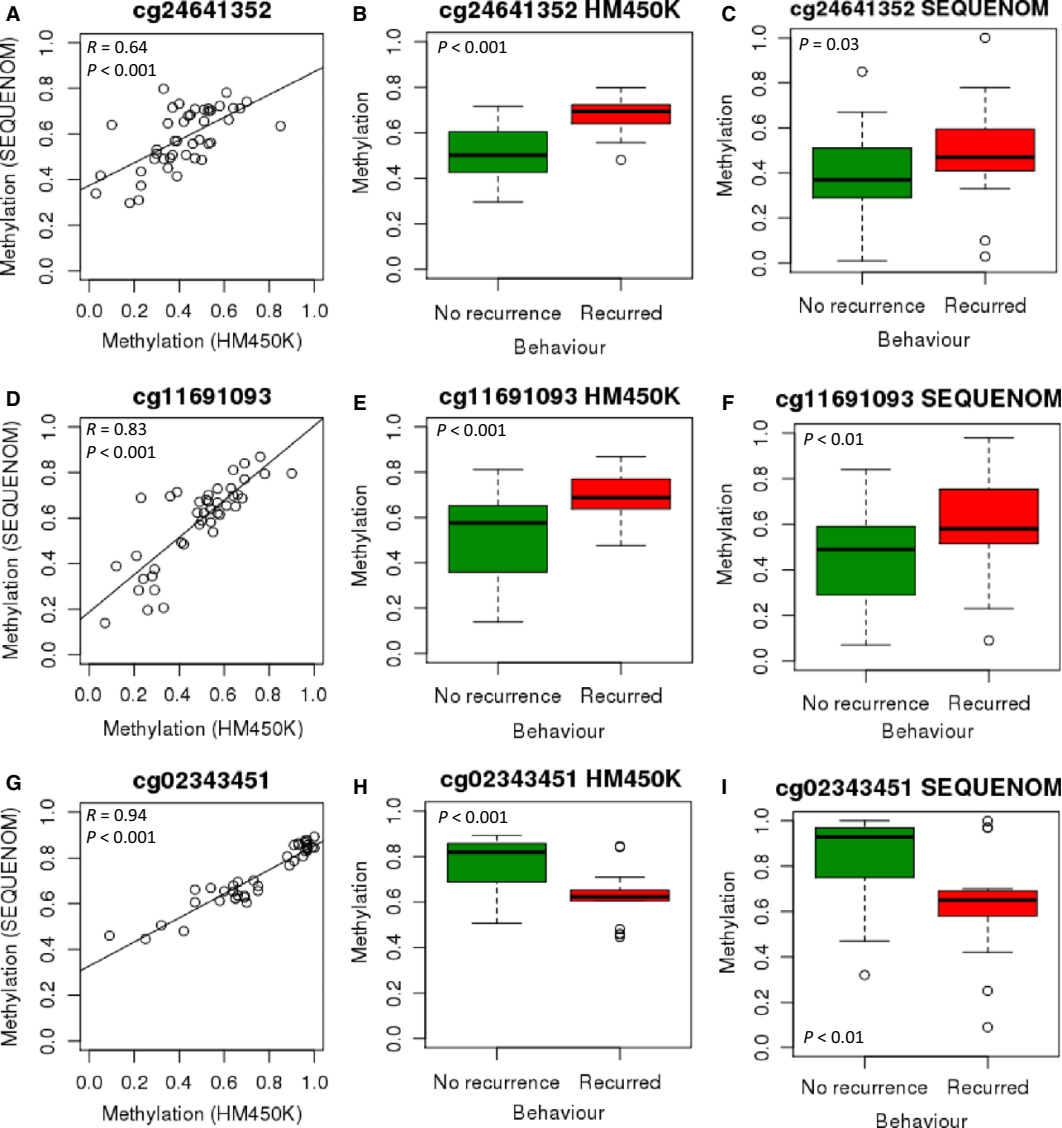
(A) Correlation between HM450K and SEQUENOM at cg24641352 (*n* = 44). (B) Median methylation at cg24641352 (HM450K) was significantly higher in tumours that recurred than in those that did not (0.69 vs 0.50, *n* = 46). (C) Median methylation at cg24641352 (SEQUENOM) was significantly higher in tumours that recurred than in those that did not (0.47 vs 0.37, *n* = 53). (D) Correlation between HM450K and SEQUENOM at cg11691093 (*n* = 42). (E) Median methylation at cg11691093 (HM450K) was significantly higher in tumours that recurred than in those that did not (0.69 vs 0.58, *n* = 46). (F) Median methylation at cg11691093 (SEQUENOM) was significantly higher in tumours that recurred than in those that did not (0.58 vs 0.49, *n* = 54). (G) Correlation between HM450K and SEQUENOM at cg02343451 (*n *= 39). (H) Median methylation at cg02343451 (HM450K) was significantly lower in tumours that recurred than in those that did not (0.62 vs 0.82, *n* = 46). (I) Median methylation at cg02343451 (SEQUENOM) was significantly lower in tumours that recurred than in those that did not (0.65 vs 0.93, *n* = 43).

Linear regression analysis identified 4935 DMPs (unadjusted *P* ≤ 0.01) associated with tumour behaviour, one of which (cg13890972) remained significant after FDR correction. Unfortunately, due to being located within a repetitive genomic region (12p11.22), this could not be validated using locus‐specific analysis. Three additional CpG sites (of low unadjusted *P*‐value) were therefore selected for verification and validation: cg24641352 (located in the transcription start site of *ZFP41*), cg11691093 (located in the gene body of *MYH9*) and cg02343451 (no gene association). Each showed a significant difference in methylation between the two behaviour groups (*P* ≤ 0.01, Fig. 6B,E,H).

Locus‐specific methylation analysis (SEQUENOM) was carried out for each of these three sites on 45 of the 46 above samples (one sample had insufficient DNA and tissue for analysis), and an additional 14 paediatric PAs from Melbourne (nine that recurred and five that did not). Results largely validated the HM450K data, with good correlations (*R* value) between the two platforms (Fig. 6A,D,G). Further, the three sites remained significantly differentially methylated between the two behaviour groups (*P* ≤ 0.05) in the replication cohort (Fig. 6C,F,I).

### 3.5 Clinical features in isolation are associated with event‐free survival time and predict tumour recurrence in paediatric PA

**Figure 7:**
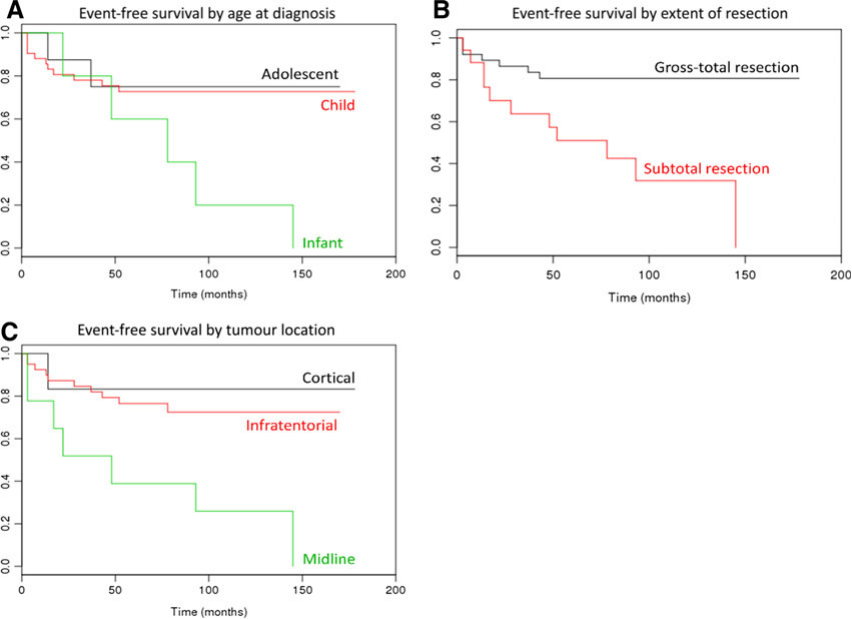
Probability of survival at any particular time point is shown on the *y*‐axis; time (months) is shown on the *x*‐axis. (A) Patients diagnosed as infants had significantly shorter EFS than those diagnosed as children (*P* ≤ 0.05). There was no significant difference in EFS between patients diagnosed as children or during adolescence, or between patients diagnosed as infants or during adolescence. (B) Patients who underwent subtotal resection had significantly shorter EFS than those who underwent gross‐total resection (*P* ≤ 0.01). (C) Patients with a tumour located in the midline had a significantly shorter EFS than those with a tumour located in the cortical or infratentorial regions (both *P* ≤ 0.05). There was no significant difference in EFS between patients with a cortical tumour as compared to those with an infratentorial tumour.

Clinical features known to be associated with poorer outcome in paediatric PA were assessed for their effect on EFS in the Melbourne cohort. As shown in Fig. 7, EFS was significantly shorter in (a) patients diagnosed as infants relative to those diagnosed in childhood, (b) those with subtotal resection as opposed to gross‐total resection and (c) those with a tumour in the midline relative to those with a cortical or infratentorial tumour (all *P* ≤ 0.05). Patient age (infant vs noninfant, AUC = 0.64), tumour location (midline vs non‐midline, AUC = 0.68) and extent of surgical resection (subtotal vs gross‐total, AUC = 0.75) all had some independent utility in classifying tumours into behaviour groups, with the highest AUC obtained by combining all three features (0.84, Table S5).

### 3.6 DNA methylation combined with clinical features increases predictive accuracy

Two of the three selected HM450K probes gave a higher AUC value than clinical features (individually or combined) in isolation, with methylation at cg02343451 alone producing the highest AUC value of 0.89 (Table S6). To extend this, ROC analysis was carried out on SEQUENOM data of each site individually and all three sites in combination.

ROC analysis identified that age, location or resection status, combined with methylation at cg02343451 (measured by HM450K), produced higher AUC values (0.90, 0.93 and 0.92, respectively) than methylation or clinical features in isolation (Table 1). The combination of resection and methylation at cg02343451 (measured by SEQUENOM) also provided a higher AUC value (0.87) than methylation features (SEQUENOM), or clinical features, in isolation (Table 1). The greatest predictive power was obtained from the combination of all three clinical features with methylation at cg02343451 (measured either by HM450K or by SEQUENOM), with AUC values of 0.95 and 0.94, respectively (Table 1).

**Table 1:** ROC analysis: utility of clinical and DNA methylation features to predict tumour recurrence

Features	HM450K		SEQUENOM	
	AUC	95% Confidence Interval	AUC	95% Confidence Interval
Age + cg24641352	0.58	0.38–0.78	0.55	0.37–0.72
Age + cg11691093	0.55	0.35–0.74	0.55	0.38–0.72
Age + cg02343451	0.90	0.80–1.00	0.79	0.63–0.95
Location + cg24641352	0.53	0.32–0.73	0.60	0.43–0.76
Location + cg11691093	0.52	0.32–0.73	0.60	0.43–0.77
Location + cg02343451	0.93	0.85–1.00	0.82	0.67–0.96
Resection + cg24641352	0.60	0.39–0.80	0.62	0.45–0.79
Resection + cg11691093	0.62	0.42–0.81	0.59	0.42–0.76
Resection + cg02343451	0.92	0.83–1.00	0.87	0.76–0.99
Age, location, resection + cg02343451	0.95	0.89–1.00	0.94	0.87–1.00

## 4 Discussion

Aberrant DNA methylation has been studied extensively in cancer and is a near ubiquitous feature of human neoplasia (Jones and Baylin, 2002). DNA methylation has the potential to provide insights into tumour biology, to identify biomarkers of disease progression and to inform novel treatment approaches (Jones and Baylin, 2002). In the current study, we have used genome‐wide DNA methylation analysis to identify location‐specific DNA methylation differences in tumours of infratentorial, midline and cortical origin and to identify a potential biomarker predictive of tumour behaviour in paediatric PA.

Histologically, PAs from different brain regions are generally indistinguishable from one another; however, their variable behaviour suggests that there may be underlying biological or tumour microenvironmental differences that contribute to disease progression (Chen and Gutmann, 2014; Gilbertson and Gutmann, 2007). It is hypothesised that glial tumours, such as PA, can arise from either mature astrocytes, radial glial cells or neural stem cells (NSCs) and that tumours in different brain regions may arise from different precursor cell populations, contributing to their variable behaviour (Chen and Gutmann, 2014; Gilbertson and Gutmann, 2007). Furthermore, it is understood that different cell types in the CNS respond differently to extrinsic factors depending on their location; for example, the loss of NF1 expression in cortical astrocytes has no effect on cell proliferation *in vitro*, whereas loss in optic nerve, cerebellar and brainstem astrocytes results in increased cell proliferation (Yeh *et al*., 2009). Additionally, cerebellum and third ventricle‐derived NSCs from the brainstem of mice expressing *KIAA1549‐BRAF* (by retroviral infection) exhibit increased proliferation, whilst NSCs from the lateral ventricle subventricular zone and neocortex do not (Kaul *et al*., 2012; Lee *et al*., 2012).

The notion that underlying biological features of paediatric PA differ by location, partly due to the cell(s) of origin, is supported by several previous studies. These have shown that histologically identical tumours have different methylation and gene expression profiles depending on their location and that these differences are associated with genes involved in nervous system development pathways (Bergthold *et al*., 2015; Lambert *et al*., 2013; Sharma *et al*., 2007; Tchoghandjian *et al*., 2009). Here, we have provided further evidence in support of this concept, identifying location‐specific differential methylation of genes involved in embryonic nervous system development, several of which have been previously reported, including *LHX2*,* NR2E1*,* SIX3*,* PAX3* and *IRX2* (Bergthold *et al*., 2015; Lambert *et al*., 2013; Sharma *et al*., 2007; Tchoghandjian *et al*., 2009). Additionally, our analysis showed that many of the location‐specific differences identified in DNA methylation profile were reflected in control adult brain, which supports the concept that tissue of origin is an important driver of DNA methylation profile of histologically similar tumours.

To further explore this, we investigated two such genes, *NEUROG1* and *NR2E1*. During embryonic development, most progenitor cells in the CNS can generate neurons, astrocytes and oligodendrocytes, with cell‐fate specification controlled by a number of molecules including *NEUROG1* (Gilbertson and Gutmann, 2007; Ma *et al*., 1997). *NEUROG1* is a proneural transcription factor that inhibits differentiation of NSCs into astrocytes (Sun *et al*., 2001). *Neurog1* is also expressed in the developing rodent CNS adjacent to, and in a nonoverlapping pattern with, another neuronal development gene *Mash1*, which suggests that it is involved in the development of location‐specific NSC populations (Ma *et al*., 1997). We found significantly higher methylation in the transcription start site of *NEUROG1* in cortical tumours when compared with both midline and infratentorial tumours. Midline tumours also had significantly higher methylation than infratentorial tumours. In combination, these findings suggest that PAs from different locations may develop from progenitor cells with different location‐specific genetic and epigenetic profiles.

NSCs are present in the adult brain, but remain in an inactive nondividing state (Niu *et al*., 2011). The nuclear receptor tailless (*NR2E1*) acts as a key regulator of NSC expansion during fetal forebrain development and maintains NSCs in the adult brain (Park *et al*., 2010; Sharma *et al*., 2007). Recently, it has been demonstrated that *Nr2e1* expression can activate postnatal NSCs in young mice (Niu *et al*., 2011), whereas overexpression in adult mice leads to a migration of NSCs from their natural niche, to the production of neurons and to the development of gliomas (Liu *et al*., 2010). In humans, increased *NR2E1* expression has been reported in glioma cell lines and glial tumours including astrocytomas (Liu *et al*., 2010). Overexpression is correlated with poorer survival in patients with glioma (Park *et al*., 2010). In this study, we identified *NR2E1* to have significantly higher gene body methylation in cortical relative to midline and infratentorial tumours. Midline tumours also had significantly higher methylation than infratentorial tumours. This confirms findings from work by Lambert *et al*. who also identified hypermethylation in the gene body of *NR2E1* in supratentorial tumours in comparison with infratentorial tumours. This was associated with significantly higher *NR2E1* expression (Lambert *et al*., 2013), in accordance with an earlier study that identified ninefold higher expression of *NR2E1* in supratentorial in comparison with infratentorial PAs (Sharma *et al*., 2007). Despite the previous work showing differential expression depending on tumour location, in the current study we showed variable expression of the NR2E1 protein across all tumours irrespective of location, as well as within tumours. This was not apparent in control tissue (normal cortex); therefore, it is unlikely to be a technical artefact and is consistent with dysregulation of expression of NR2E1 in PA. These contrasting findings require further investigation in order to better understand the role of *NR2E1* in the development of PA.

An important aspect in the treatment of paediatric PA is managing tumour recurrence, associated with extracerebellar tumour location, subtotal surgical resection and poorer outcomes (Armstrong *et al*., 2009; Bandopadhayay *et al*., 2014; Collins *et al*., 2015; Dodgshun *et al*., 2016; Fernandez *et al*., 2003; Krishnatry *et al*., 2016; Lund *et al*., 2011; Ohgaki and Kleihues, 2005; Youland *et al*., 2013). At present, there are limited data related to the molecular features underpinning this behaviour. Although we were unable to elucidate any differentially methylated biological networks between recurring and nonrecurring tumours, we were able to define an accurate method to classify tumours into behaviour groups at diagnosis by combining patient age, tumour location, extent of resection and DNA methylation at a specific CpG site. This is the first report demonstrating the utility of a molecular feature in predicting recurrence in paediatric PA and has potential implications for clinical management particularly selection of surveillance and treatment protocols.

## 5 Conclusion

In summary, this study has identified distinct location‐specific DNA methylation profiles in a large cohort of paediatric PA, indicating that despite their often identical histology, they have different underlying location‐specific epigenetic properties potentially determined by the progenitor cells from which they develop (Gilbertson and Gutmann, 2007). Further in‐depth analysis of epigenetic, genetic, gene expression and tumour microenvironmental factors will help to further characterise the progenitor cell(s) of PA, potentially explaining their behaviour. Importantly, whilst previous studies have explored differences between tumours at the level of the three‐vesicle stage of development, forebrain (supratentorium, hypothalamus/chiasm) and hindbrain (infratentorium/posterior fossa), in the current study we have explored differences between tumours at the level of the five‐vesicle stage of development, the telencephalon (cortical/cerebral hemisphere), diencephalon (midline supratentorial structures including thalamus and optic nerve and tract) and metencephalon (infratentorium; Kandel *et al*., 2000). Grouping tumours in this way allows investigation of supratentorial midline tumours separately to those in the cerebral cortex, a clinically relevant comparison considering the difficulty of surgical resection and high rates of recurrence in midline tumours (Tchoghandjian *et al*., 2009). Furthermore, the study also identified a highly accurate method to classify tumours into behaviour groups at diagnosis. This provides a basis from which to conduct future studies, applying more powerful machine‐based learning approaches to DNA methylation profiles in a larger tumour series, and offers considerable promise to more fully explore the potential of methylation as a predictive tool in paediatric CNS (and other) tumours.

## Ethics approval

This study was approved by the Royal Children's Hospital Human Research Ethics Committee, HREC 34040A and 34049C.

## Data accessibility

The data sets used during the current study are available from the corresponding author on reasonable request.

## Author contributions

ASO contributed to the conception and design of the study, acquired data from the Melbourne cohort, analysed and interpreted data from both the Melbourne and Cambridge/Heidelberg cohorts and drafted the manuscript. AD contributed to drafting the manuscript. VH and DTWJ provided the data and associated clinical information for the Cambridge/Heidelberg cohort and reviewed the manuscript critically. DMA reviewed the manuscript critically. MS contributed to drafting the manuscript. DM reviewed all tissue slides from the Melbourne cohort. RS was a major contributor in drafting the manuscript and made a substantial contribution to the conception and design of the study. All authors read and approved the final manuscript.

## Supporting information


**Table S1.** Primer sequences for locus‐specific DNA methylation detection.
**Table S2.** Primer sequences for *BRAF* V600E mutation detection.
**Table S3.** Study cohort: tumour location analysis.
**Table S4.** Study cohort: tumour behaviour analysis.
**Table S5.** ROC analysis: utility of clinical features to predict tumour recurrence.
**Table S6.** ROC analysis: utility of DNA methylation biomarkers to predict tumour recurrence.

## References

[mol212062-bib-0001] Armstrong GT , Liu Q , Yasui Y , Huang S , Ness KK , Leisenring W , Hudson MM , Donaldson SS , King AA and Stovall M (2009) Long‐term outcomes among adult survivors of childhood central nervous system malignancies in the Childhood Cancer Survivor Study. J Natl Cancer Inst 101, 946–958. 19535780 10.1093/jnci/djp148 PMC2704230

[mol212062-bib-0002] Bandopadhayay P , Bergthold G , London WB , Goumnerova LC , Morales La Madrid A , Marcus KJ , Guo D , Ullrich NJ , Robison NJ and Chi SN (2014) Long‐term outcome of 4,040 children diagnosed with pediatric low‐grade gliomas: an analysis of the Surveillance Epidemiology and End Results (SEER) database. Pediatr Blood Cancer 61, 1173–1179. 24482038 10.1002/pbc.24958 PMC4657506

[mol212062-bib-0003] Benjamini Y and Hochberg Y (1995) Controlling the false discovery rate: a practical and powerful approach to multiple testing. J R Stat Soc B Meth 57, 289–300.

[mol212062-bib-0004] Bergthold G , Bandopadhayay P , Hoshida Y , Ramkissoon S , Ramkissoon L , Rich B , Maire CL , Paolella BR , Schumacher SE and Tabak B (2015) Expression profiles of 151 pediatric low‐grade gliomas reveal molecular differences associated with location and histological subtype. Neuro‐oncology 17, 1486–1496. 25825052 10.1093/neuonc/nov045 PMC4648300

[mol212062-bib-0005] Burkhard C , Di Patre P‐L , Schüler D , Schüler G , Yasargil MG , Yonekawa Y , Lütolf UM , Kleihues P and Ohgaki H (2003) A population‐based study of the incidence and survival rates in patients with pilocytic astrocytoma. J Neurosurg 98, 1170–1174. 12816259 10.3171/jns.2003.98.6.1170

[mol212062-bib-0006] Chen Y and Gutmann D (2014) The molecular and cell biology of pediatric low‐grade gliomas. Oncogene 33, 2019–2026. 23624918 10.1038/onc.2013.148

[mol212062-bib-0007] Collins VP , Jones DT and Giannini C (2015) Pilocytic astrocytoma: pathology, molecular mechanisms and markers. Acta Neuropathol 129, 775–788. 25792358 10.1007/s00401-015-1410-7 PMC4436848

[mol212062-bib-0008] Dodgshun AJ , Maixner WJ , Hansford JR and Sullivan MJ (2016) Low rates of recurrence and slow progression of pediatric pilocytic astrocytoma after gross‐total resection: justification for reducing surveillance imaging. J Neurosurg Pediatr 17, 569–572. 26722760 10.3171/2015.9.PEDS15449

[mol212062-bib-0009] Fernandez C , Figarella‐Branger D , Girard N , Bouvier‐Labit C , Gouvernet J , Paredes AP and Lena G (2003) Pilocytic astrocytomas in children: prognostic factors—a retrospective study of 80 cases. Neurosurgery 53, 544–555. 12943571 10.1227/01.neu.0000079330.01541.6e

[mol212062-bib-0010] Gilbertson RJ and Gutmann DH (2007) Tumorigenesis in the brain: location, location, location. Cancer Res 67, 5579–5582. 17575119 10.1158/0008-5472.CAN-07-0760

[mol212062-bib-0011] Gnekow AK , Falkenstein F , von Hornstein S , Zwiener I , Berkefeld S , Bison B , Warmuth‐Metz M , Driever PH , Soerensen N , Kortmann R‐D *et al* (2012) Long‐term follow‐up of the multicenter, multidisciplinary treatment study HIT‐LGG‐1996 for low‐grade glioma in children and adolescents of the German Speaking Society of Pediatric Oncology and Hematology. Neuro‐oncology 14, 1265–1284. 22942186 10.1093/neuonc/nos202 PMC3452343

[mol212062-bib-0012] Horbinski C (2013) To BRAF or not to BRAF: is that even a question anymore? J Neuropath Exp Neur 72, 2–7. 23242278 10.1097/NEN.0b013e318279f3db PMC3530158

[mol212062-bib-0013] Jones PA and Baylin SB (2002) The fundamental role of epigenetic events in cancer. Nat Rev Genet 3, 415–428. 12042769 10.1038/nrg816

[mol212062-bib-0014] Jones DT , Ichimura K , Liu L , Pearson DM , Plant K and Collins VP (2006) Genomic analysis of pilocytic astrocytomas at 0.97 Mb resolution shows an increasing tendency toward chromosomal copy number change with age. J Neuropath Exp Neur 65, 1049–1058. 17086101 10.1097/01.jnen.0000240465.33628.87 PMC2761618

[mol212062-bib-0015] Kandel E , Schwartz J and Jessell T (2000) Principles of Neural Science, 4th edn McGraw‐Hill, New York.

[mol212062-bib-0016] Kaul A , Chen Y‐H , Emnett RJ , Dahiya S and Gutmann DH (2012) Pediatric glioma‐associated KIAA1549: BRAF expression regulates neuroglial cell growth in a cell type‐specific and mTOR‐dependent manner. Gene Dev 26, 2561–2566. 23152448 10.1101/gad.200907.112 PMC3521628

[mol212062-bib-0017] Krishnatry R , Zhukova N , Guerreiro Stucklin AS , Pole JD , Mistry M , Fried I , Ramaswamy V , Bartels U , Huang A and Laperriere N (2016) Clinical and treatment factors determining long‐term outcomes for adult survivors of childhood low‐grade glioma: a population‐based study. Cancer 122, 1261–1269. 26970559 10.1002/cncr.29907

[mol212062-bib-0018] Lambert SR , Witt H , Hovestadt V , Zucknick M , Kool M , Pearson DM , Korshunov A , Ryzhova M , Ichimura K and Jabado N (2013) Differential expression and methylation of brain developmental genes define location‐specific subsets of pilocytic astrocytoma. Acta Neuropathol 126, 291–301. 23660940 10.1007/s00401-013-1124-7

[mol212062-bib-0019] Lee DY , Gianino SM and Gutmann DH (2012) Innate neural stem cell heterogeneity determines the patterning of glioma formation in children. Cancer Cell 22, 131–138. 22789544 10.1016/j.ccr.2012.05.036 PMC3396885

[mol212062-bib-0020] Liu H‐K , Wang Y , Belz T , Bock D , Takacs A , Radlwimmer B , Barbus S , Reifenberger G , Lichter P and Schütz G (2010) The nuclear receptor tailless induces long‐term neural stem cell expansion and brain tumor initiation. Gene Dev 24, 683–695. 20360385 10.1101/gad.560310 PMC2849125

[mol212062-bib-0021] Lund LW , Schmiegelow K , Rechnitzer C and Johansen C (2011) A systematic review of studies on psychosocial late effects of childhood cancer: structures of society and methodological pitfalls may challenge the conclusions. Pediatr Blood Cancer 56, 532–543. 21298737 10.1002/pbc.22883

[mol212062-bib-0022] Ma Q , Sommer L , Cserjesi P and Anderson DJ (1997) Mash1 and neurogenin1 expression patterns define complementary domains of neuroepithelium in the developing CNS and are correlated with regions expressing notch ligands. J Neurosci 17, 3644–3652. 9133387 10.1523/JNEUROSCI.17-10-03644.1997 PMC6573688

[mol212062-bib-0023] Maksimovic J , Gordon L and Oshlack A (2012) SWAN: subset‐quantile within array normalization for illumina infinium HumanMethylation450 BeadChips. Genome Biol 13, 44–56. 10.1186/gb-2012-13-6-r44 PMC3446316 22703947

[mol212062-bib-0024] Marko NF and Weil RJ (2012) The molecular biology of WHO grade I astrocytomas. Neuro‐oncology 14, 1424–1431. 23090984 10.1093/neuonc/nos257 PMC3499013

[mol212062-bib-0025] Myung JK , Cho H , Park C‐K , Kim S‐K , Lee S‐H , Park S‐H (2012) Analysis of the BRAFV600E mutation in central nervous system tumors. Transl Oncol 5, 430–436. 23323158 10.1593/tlo.12328 PMC3542839

[mol212062-bib-0026] Niu W , Zou Y , Shen C and Zhang C‐L (2011) Activation of postnatal neural stem cells requires nuclear receptor TLX. J Neurosci 31, 13816–13828. 21957244 10.1523/JNEUROSCI.1038-11.2011 PMC3192402

[mol212062-bib-0027] Northcott PA , Pfister SM and Jones DT (2015) Next‐generation (epi)genetic drivers of childhood brain tumours and the outlook for targeted therapies. Lancet Oncol 16, e293–e302. 26065614 10.1016/S1470-2045(14)71206-9

[mol212062-bib-0028] Ohgaki H and Kleihues P (2005) Population‐based studies on incidence, survival rates, and genetic alterations in astrocytic and oligodendroglial gliomas. J Neuropath Exp Neur 64, 479–489. 15977639 10.1093/jnen/64.6.479

[mol212062-bib-0029] Ollikainen M , Smith KR , Joo EJ‐H , Ng HK , Andronikos R , Novakovic B , Aziz NKA , Carlin JB , Morley R and Saffery R (2010) DNA methylation analysis of multiple tissues from newborn twins reveals both genetic and intrauterine components to variation in the human neonatal epigenome. Hum Mol Genet 19, 4176–4188. 20699328 10.1093/hmg/ddq336

[mol212062-bib-0030] Ostrom QT , Gittleman H , Fulop J , Liu M , Blanda R , Kromer C , Wolinsky Y , Kruchko C , Barnholtz‐Sloan JS (2015) CBTRUS statistical report: primary brain and central nervous system tumors diagnosed in the United States in 2008‐2012. Neuro‐oncology 17, iv1–iv62. 26511214 10.1093/neuonc/nov189 PMC4623240

[mol212062-bib-0031] Pajtler KW , Witt H , Sill M , Jones DT , Hovestadt V , Kratochwil F , Wani K , Tatevossian R , Punchihewa C and Johann P (2015) Molecular classification of ependymal tumors across all CNS compartments, histopathological grades, and age groups. Cancer Cell 27, 728–743. 25965575 10.1016/j.ccell.2015.04.002 PMC4712639

[mol212062-bib-0032] Park H‐J , Kim J‐K , Jeon H‐M , Oh S‐Y , Kim S‐H , Park M‐J , Soeda A , Nam D‐H and Kim H (2010) The neural stem cell fate determinant TLX promotes tumorigenesis and genesis of cells resembling glioma stem cells. Mol Cells 30, 403–408. 20814749 10.1007/s10059-010-0122-z

[mol212062-bib-0033] Pfister S , Janzarik WG , Remke M , Ernst A , Werft W , Becker N , Toedt G , Wittmann A , Kratz C and Olbrich H (2008) BRAF gene duplication constitutes a mechanism of MAPK pathway activation in low‐grade astrocytomas. J Clin Invest 118, 1739–1749. 18398503 10.1172/JCI33656 PMC2289793

[mol212062-bib-0034] Sharma MK , Mansur DB , Reifenberger G , Perry A , Leonard JR , Aldape KD , Albin MG , Emnett RJ , Loeser S and Watson MA (2007) Distinct genetic signatures among pilocytic astrocytomas relate to their brain region origin. Cancer Res 67, 890–900. 17283119 10.1158/0008-5472.CAN-06-0973

[mol212062-bib-0035] Sturm D , Witt H , Hovestadt V , Khuong‐Quang DA , Jones DT , Konermann C , Pfaff E , Tonjes M , Sill M , Bender S *et al* (2012) Hotspot mutations in H3F3A and IDH1 define distinct epigenetic and biological subgroups of glioblastoma. Cancer Cell 22, 425–437. 23079654 10.1016/j.ccr.2012.08.024

[mol212062-bib-0036] Sun Y , Nadal‐Vicens M , Misono S , Lin MZ , Zubiaga A , Hua X , Fan G and Greenberg ME (2001) Neurogenin promotes neurogenesis and inhibits glial differentiation by independent mechanisms. Cell 104, 365–376. 11239394 10.1016/s0092-8674(01)00224-0

[mol212062-bib-0037] Tchoghandjian A , Fernandez C , Colin C , El Ayachi I , Voutsinos‐Porche B , Fina F , Scavarda D , Piercecchi‐Marti M‐D , Intagliata D and Ouafik LH (2009) Pilocytic astrocytoma of the optic pathway: a tumour deriving from radial glia cells with a specific gene signature. Brain 132, 1523–1535. 19336457 10.1093/brain/awp048

[mol212062-bib-0038] Wiestler B , Capper D , Sill M , Jones DT , Hovestadt V , Sturm D , Koelsche C , Bertoni A , Schweizer L and Korshunov A (2014) Integrated DNA methylation and copy‐number profiling identify three clinically and biologically relevant groups of anaplastic glioma. Acta Neuropathol 128, 561–571. 25008768 10.1007/s00401-014-1315-x

[mol212062-bib-0039] Yeh TH , Lee DY , Gianino SM and Gutmann DH (2009) Microarray analyses reveal regional astrocyte heterogeneity with implications for neurofibromatosis type 1 (NF1)‐regulated glial proliferation. Glia 57, 1239–1249. 19191334 10.1002/glia.20845 PMC2706934

[mol212062-bib-0040] Youland RS , Khwaja SS , Schomas DA , Keating GF , Wetjen NM and Laack NN (2013) Prognostic factors and survival patterns in pediatric low‐grade gliomas over 4 decades. J Pediatr Hematol Oncol 35, 197–205. 22983418 10.1097/MPH.0b013e3182678bf8

[mol212062-bib-0041] Zhang J , Wu G , Miller CP , Tatevossian RG , Dalton JD , Tang B , Orisme W , Punchihewa C , Parker M , Qaddoumi I *et al* (2013) Whole‐genome sequencing identifies genetic alterations in pediatric low‐grade gliomas. Nat Genet 45, 602–612. 23583981 10.1038/ng.2611 PMC3727232

