# Supplementary material for: Methylation profiling of paediatric pilocytic astrocytoma reveals variants specifically associated with tumour location and predictive of recurrence
Source: Mol Oncol. 2018 Jul 6;12(8):1219–32. doi: 10.1002/1878-0261.12062 (PMC6068350; doi:10.1002/1878-0261.12062)
Supplement: Supplementary file 1 — Table S1. Primer sequences for locus‐specific DNA methylation detection. Table S2. Primer sequences for BRAF V600E mutation detection. Table S3. Study cohort: tumour location analysis. Table S4. Study cohort: tumour behaviour analysis. Table S5. ROC analysis: utility of clinical features to predict tumour recurrence. Table S6. ROC analysis: utility of DNA methylation biomarkers to predict tumour recurrence. [file MOL2-12-1219-s001.docx]

| **Supplementary Table 1. Primer sequences for locus-specific DNA methylation detection.** | | | | |
| --- | --- | --- | --- | --- |
| Target probe  (HM450K) | Genomic coordinates (hg19) | Primer sequence | Amplicon length (bp) | Annealing temperatures (°C) |
| cg24641352 | chr8:144,328,004-144,328,182 | F: GGTTGATTAGAGTTGAGGATTTAGTTAAG  R: ATACTCACTCCACTTCCTACCTACCC | 179 | 56, 60 |
| cg11691093 | chr22:36,727,290-36,727,433 | F: GAAATTTAGGGAGAAAATGGTT  R: ATATAAAACAAATACCCAAACATACC | 144 | 58, 58 |
| cg02343451 | chr16:3,418,828-3,419,007 | F: TAGAGGTTTTTGTTAGGTTTTTTGT  R: AACTAATCCCTCTAATAATTAATACACCT | 180 | 58, 60 |

| **Supplementary Table 2. Primer sequences for *BRAF* V600E mutation detection.** | | | | |
| --- | --- | --- | --- | --- |
| Target | Genomic coordinates (hg19) | Primer sequence | Amplicon length (bp) | Annealing temperature (°C) |
| *BRAF* V600E | chr 7: 140,453,013-140,453,259 | F: GCTTGCTCTGATAGGAAAATGAG  R: GTAACTCAGCAGCATCTCAGG | 237 | 62 |

| Supplementary Table 3. Study cohort: tumour location analysis. | | |
| --- | --- | --- |
| Clinical Characteristics | n | % |
| Sex |  |  |
| Female | 57 | 48.7 |
| Male | 60 | 51.3 |
| Age at diagnosis |  |  |
| Infant (0-18 months) | 8 | 6.8 |
| Child (19 months-11 years) | 87 | 74.4 |
| Adolescent (12-18 years) | 22 | 18.8 |
| Broad tumour location  Infratentorial  Midline (supratentorial)  Cortical | 82  23  12 | 70.0  19.7  10.3 |
| Extent of surgical resection |  |  |
| Gross total resection | 60 | 51.3 |
| Subtotal resection | 37 | 31.6 |
| Unknown | 20 | 17.1 |
| Other medical conditions |  |  |
| NF1 | 3 | 2.6 |
| *BRAF* status |  |  |
| Any fusion  Any mutation | 83  8 | 70.9  6.8 |
| Patient outcome |  |  |
| ≥5 year overall survival | 80 | 68.4 |
| Deceased | 6 | 5.2 |
| Unknown (<5 years of follow up) | 31 | 26.5 |

| Supplementary Table 4. Study cohort: tumour behaviour analysis. | | |
| --- | --- | --- |
| Clinical Characteristics | Recurred (18)  n (%) | No Recurrence (28)  n (%) |
| Sex  Female  Male | 10 (55.6)  8 (44.4) | 17 (60.7)  11 (39.3) |
| Age at diagnosis  Infant (0-18 months)  Child (19 months-11 years)  Adolescent (12-18 years) | 5 (27.8)  11 (61.1)  2 (11.1) | 0  24 (85.7)  4 (14.3) |
| Tumour location  Infratentorial  Midline (supratentorial)  Cortical | 10 (55.6)  7 (38.9)  1 (5.6) | 22 (78.6)  1 (3.6)  5 (17.9) |
| Extent of surgical resection  Gross total resection  Subtotal resection  Biopsy only | 7 (38.9)  8 (44.4)  3 (16.7) | 25 (89.3)  2 (7.1)  1 (3.6) |
| Adjuvant treatment  Chemotherapy and radiotherapy  Chemotherapy only  No treatment | 4 (22.2)  8 (44.4)  6 (33.3) | 0  2 (7.1)  26 (92.9) |
| Other medical conditions  NF1 | 0 | 0 |
| *KIAA1549-BRAF* status  Fusion  No fusion | 9 (50)  9 (50) | 18 (64.3)  10 (36) |
| Outcome  Alive  Deceased | 14 (77.8)  4 (22.2) | 28 (100)  0 |

| Supplementary Table 5. ROC analysis: utility of clinical features to predict rumour recurrence. | | |
| --- | --- | --- |
| Feature | AUC | 95% Confidence Interval |
| Age at diagnosis (infant vs non-infant) | 0.64 | 0.53-0.75 |
| Location (midline vs non-midline) | 0.68 | 0.56-0.80 |
| Surgical resection (subtotal vs gross-total) | 0.75 | 0.62-0.88 |
| Age + Location | 0.71 | 0.59-0.83 |
| Age + Surgery | 0.79 | 0.67-0.92 |
| Location + Surgery | 0.83 | 0.72-0.95 |
| Age, location and surgical resection | 0.84 | 0.73-0.96 |

| Supplementary Table 6. ROC analysis: utility of DNA methylation biomarkers to predict tumour recurrence. | | | | | |
| --- | --- | --- | --- | --- | --- |
|  | HM450K | | SEQUENOM | |  |
| Probe ID | AUC | 95% Confidence Interval | AUC | 95% Confidence Interval | ∆ AUC |
| cg24641352 | 0.84 | 0.72-0.95 | 0.67 | 0.52-0.82 | 0.17 |
| cg11691093 | 0.81 | 0.69-0.94 | 0.71 | 0.56-0.85 | 0.10 |
| cg02343451 | 0.89 | 0.79-1.00 | 0.76 | 0.59-0.92 | 0.13 |
| cg24641352 + cg11691093 | 0.89 | 0.79-0.89 | 0.80 | 0.67-0.93 | 0.09 |
| cg24641352 + cg02343451 | 0.51 | 0.34-0.69 | 0.69 | 0.52-0.87 | -0.18 |
| cg11691093 + cg02343451 | 0.49 | 0.32-0.66 | 0.63 | 0.45-0.81 | -0.14 |
| All three probes combined | 0.71 | 0.55-0.86 | 0.53 | 0.34-0.71 | 0.18 |
